# Travels through Different Provinces of the Russian Empire

**Published:** 1781-03

**Authors:** 


III. *Travels through different Provinces of the Russian Empire, by* P. S. Pallas, M. D. &c. (Continued from page 321.) Vol. II. 744 pages, with 35 copper-plates. Vol. III. 102 pages, with 53 copper-plates.

IN the second and third volumes, Dr. Pallas gives an account of his travels through different parts of Siberia. In the open fields in Isetkiy he found *valeriana phu* growing in great plenty; this plant is much used by the common people as a remedy for epilepsy and convulsions in children.

They likewiſe employ a fungus that grows on the larch tree, as a cure for agues and fluor albus.

The Barſchkirs employ the bulbous root of the *lilium martagon*, either raw or dried, as an article of food in winter.

On the road to Tſcherno-Iſtotſchinſkoi, our author obſerved that the horſes devoured the *veratrum album* with great eagerneſs. The plant was not in flower; the only effect it produced was a ſlight purging; this ſeemed the more remarkable, as it is generally ſuppoſed to prove poiſonous to animals.

In the northern parts of Siberia, the *Daphne mezerreum* is employed as a puke for children in caſes of cough; the inhabitants rub the leaves of the plant on their cheeks in their bathing rooms in order to excite a ſlight degree of redneſs in the ſkin; the berries are powdered and taken as a purge; thirty berries are a common doſe for an adult. The root, which is the moſt acrid part of the plant, is made uſe of in the tooth-ach.

Dr. Pallas ſpeaks of an epidemic that ſometimes prevails among horſes in the province of Iſetky; he aſcribes it to the *furia infernalis*, a ſmall inſect that penetrates the ſkin. The inhabitants of of that province are likewiſe ſubject to a ſimilar diſeaſe; it begins with a ſenſation of itching, which is ſucceeded by a ſmall hard tumour, not unlike that produced by the bite of a gnat; this tumour ſoon grows harder and larger, and the part becoming inſenſible, there appears a red or bluiſh ſpot, which in a ſhort time becomes gangrenous. On the river Irtiſh, the remedies employed for the cure of this ſingular diſtemper are a lixivium of the aſhes of wormwood, or a decoction of tobacco, combined with ſal ammoniacum or alum.

Our author deſcribes a new ſpecies of ſhrub, the *pterococcus aphyllus*, the freſh root of which, cut tranſverſely, yields a great quantity of a clear gum reſembling gum tragacanth.

Speaking of the *rhaponticum*, Dr. Pallas informs us that the beſt ſort comes from Udinſk, where it is procured from the *rheum undulatum*, and from another ſpecies ſomewhat different from the latter, though nearly related to it.

The Mongal Tartars, we are told, uſe the dried leaves of the *ſaxifraga craſſifolia* as a ſubſtitute for tea. The infuſion has a colour and taſte not unlike the worſt kind of bohea tea. The freſh leaves are too bitter and ſtyptic for this purpoſe.

Between Tomſk and Kraſnojarſk, near the river Ki, and likewiſe on the river Jeniſey, our author met with the ſmalleſt quadruped hitherto known. It is a ſpecies of *ſorex*, but not that deſcribed by Laxmann under the name of *ſorex minutus*; it weighs only about half a drachm, and is of a ſomewhat browner colour than the common ſorex.

As an inſtance of the great cold that prevails in Siberia during the winter months, we are told that at Kraſnojarſk, where our author wintered in 1771, the mercury in Fahrenheit's thermometer was for ſome time ſtationary at 42 degrees below 0.

Theſe two volumes being chiefly employed on natural hiſtory, it would be foreign to the plan of our work to give a minute account of their contents, we ſhall therefore only add, that the reader will find in them deſcriptions of a great variety of new animals, birds, and plants, all which are illuſtrated by ſuitable engravings.

In the ſecond volume Dr. Pallas has inſerted the obſervations made by Mr. Sokolof, an ingenious young naturaliſt, who accompanied him in his travels, and who viſited ſeveral places without our author. Mr. Sokolof's papers, beſides other intereſting matter, contain an account of the the Caſpian fiſhery, and a deſcription of the ſalt lakes at Gurjef, and of thoſe of the province of Iſetky.

